# P-139. Prophylactic Antibiotics for Transcatheter Aortic Valve Implantation (TAVI) at a UK Cardiothoracic Centre

**DOI:** 10.1093/ofid/ofaf695.366

**Published:** 2026-01-11

**Authors:** Sajeevan Rasanantham, Alexander Martin, Monika Kalra, Csaba Marodi, Igor Kubelka

**Affiliations:** South Tees NHS Foundation trust, Middlesbrough, England, United Kingdom; James Cook University Hospital, Middlesbrough, England, United Kingdom; James Cook University Hospital, Middlesbrough, England, United Kingdom; James Cook University Hospital, Middlesbrough, England, United Kingdom; James Cook University Hospital, Middlesbrough, England, United Kingdom

## Abstract

**Background:**

TAVI is an increasingly used alternative to surgical aortic valve replacement. Infective endocarditis post TAVI, while uncommon, is a serious complication, with *Enterococcus spp.* frquently implicated. In 2023, the European Society of Cardiology(ESC) issued updated Endocarditis guidelines recommending prophylaxis with activity against *Enterococcus spp. prior to TAVI(Delgado teal., Eur Heart J,* 2023). Local guidance at our institution had not previously addressed this.

**Figure 1:**
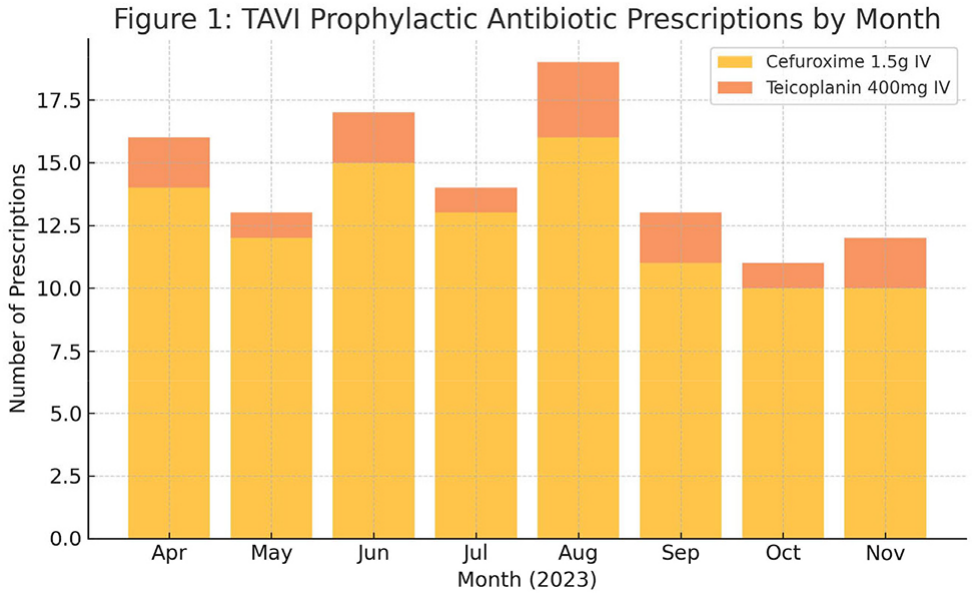
Antibioitic prescriptions for TAVI

**Methods:**

A retrospective review of electronic antibiotic prescriptions for TAVI was performed at James Cook University Hospital between April and November 2023. Cases of TAVI-related Endocarditis from January 2022 to January 2024 were identified via IE MDT records and ICD-coded hospital data. Findings were reviewed with microbiology, infectious diseases, and cardiology services.

**Results:**

Among 115 prescriptions for 112 patients, 101(88%) received cefuroxime 1.5g IV monotherapy, and 14(12%) received Teicoplanin 400mg IV monotherapy. Only Teicoplanin provided Enterococcus coverage. No patient received prophylaxis aliged with ESC or local guidance. Three cases of TAVI-related IE were identified: *Enterococcus faecalis, Streptococcus oxalis, and Streptococcus gordonii.* These findings informed he development of a revised prophylactic regimen incorporating specific coverage for Enterococcus spp., agreed upon through multidisciplinary collaboration.

**Conclusion:**

At our centre, the most patients undergoing TAVI did not receive prophylaxis with appropriate Enterococcus spp coverage, falling short of current ESC guideline recommendations. These findings prompted the development and implementation of a new institutional guideline, which will be monitered through prospective study. Addtionally, there is a pressing need for a dedicated TAVI endocarditis database to inform national and international prophylaxis strategies based on robust epidemiological data.

**Disclosures:**

All Authors: No reported disclosures

